# Cord blood mesenchymal stromal cells combined with hyaluronic acid show symptom-modifying and potential disease-modifying effects in an equine osteoarthritis fetlock chip model

**DOI:** 10.3389/fvets.2026.1896354

**Published:** 2026-08-06

**Authors:** Rodrigo M. Luque, Narman Mortagy, Terence C. McCorkell, Thomas G. Koch, Sahar Mehrpouyan, Amir Hamed Alizadeh, Keith A. Russell, Janet Beeler-Marfisi, Laurie R. Goodrich, Gabrielle Monteith, Alexander Valverde, Howard Dobson, Alex Zur Linden, Andres F. Giraldo, Judith B. Koenig

**Affiliations:** 1 Department of Biomedical Sciences, Ontario Veterinary College, University of Guelph, Guelph, ON, Canada; 2 eQcell Inc, Guelph, ON, Canada; 3 Department of Pathobiology, Ontario Veterinary College, University of Guelph, Guelph, ON, Canada; 4 Orthopaedic Research Center, Translational Medicine Institute, Colorado State University, Fort Collins, CO, United States; 5 Department of Clinical Studies, Ontario Veterinary College, University of Guelph, Guelph, ON, Canada

**Keywords:** allogeneic, equine, MSC, osteoarthritis, stromal, WORMS

## Abstract

**Introduction:**

Osteoarthritis (OA) is a major cause of lameness in horses, and current therapies largely address clinical signs rather than disease progression. Mesenchymal stromal cells (MSCs) have shown promise as disease-modifying biologics, but their effects on joint structure and immunologic phenotype remain incompletely characterized.

**Objectives:**

To evaluate the safety and efficacy of pooled cryopreserved equine umbilical cord blood–derived MSCs (eCB-MSCs) combined with hyaluronic acid (HA) for surgically induced fetlock OA in horses, using integrated clinical, imaging, and synovial biomarker outcomes.

**Study design:**

Prospective randomized experimental study.

**Methods:**

Twelve Standardbred horses underwent arthroscopic creation of an osteochondral (OC) fragment on the proximal phalanx (P1) in one metacarpophalangeal joint and were randomized to receive intra-articular eCB-MSC + HA or saline 6 weeks after surgery. At 12 weeks, a second surgery enabled joint sampling and arthroscopic scoring. Clinical lameness was assessed using subjective scoring and inertial sensor-based gait analysis. Structural progression was evaluated using radiography and high-field MRI with a modified Whole-Organ Magnetic Resonance Imaging Score (WORMS). Synovial fluid cytokines and chemokines were quantified longitudinally. Treatment effects and interactions with time-of-treatment inflammatory status and fragment size were analyzed using mixed models and non-parametric methods.

**Results:**

Both treatments were well tolerated with no clinically relevant adverse joint reactions. eCB-MSC + HA treated horses showed significantly greater improvement in both objective and subjective lameness scores than saline-treated controls. MRI showed stabilization or improvement of WORMS scores in eCB-MSC + HA treated joints, whereas saline-treated joints exhibited structural deterioration over time; radiographic and arthroscopic scores did not differ significantly between groups. Synovial cytokine responses were baseline- and fragment-dependent, with attenuation of pro-inflammatory mediators (including IL-1β and TNF-*α*) and preservation of regulatory immune signaling in eCB-MSC + HA treated joints. Pre-treatment WORMS scores were associated with multiple cytokines.

**Conclusion:**

eCB-MSC + HA therapy was safe and was associated with improved clinical signs, MRI-detected structural benefits, and immunomodulatory effects. Because the between group structural difference was significant on MRI but not on radiography or arthroscopy, and given the small sample size and short follow-up, these results suggest a symptom-modifying and potential disease-modifying role for eCB-MSC + HA in early OA, pending confirmation in larger, longer-term studies.

## 1 Introduction

OA is a debilitating condition affecting both athletes and the general population in humans and horses. Globally, human OA cases increased by 132.2% over the last three decades and are projected to rise by a further 48.6–95.1% by 2050, depending on the joint affected (1). Not only is the aging population prone to developing OA, but also former athletes have a prevalence of 59% in distal limb joints (2). Approximately 50% of horses above 15 years have OA (3), a pathology that is regarded as the cause of 60% of equine lameness based on surveys (4).

Surgical OA models are used to evaluate symptom-modifying and disease-modifying therapies. Large-animal models such as the horse offer greater translational relevance than small animals, in part because joint structure and cartilage thickness more closely approximate those of humans. Although chemically induced models reproduce key inflammatory and molecular changes, surgical models are increasingly favored because they introduce a defined biomechanical injury and tend to yield more consistent, progressive disease (5–7).

In horses, osteochondral (OC) fragment models are widely used to study OA and evaluate therapeutics. The classic carpal OC fragment-exercise model developed at Colorado State University has been applied extensively, although clinical OA is frequently observed in the metacarpophalangeal joint (8–10). To better reflect this clinical presentation, fetlock models have been developed in which an OC fragment is created arthroscopically on the proximal dorsal aspect of the P1 (11–13). In line with human classification frameworks, these models are best viewed as controlled representations of PTOA, which differs from primary OA in typical onset, multi-tissue involvement, imaging and molecular features, treatment responses, and joint mechanics (14–16).

As OA research shifts toward disease-modifying therapies, biologics have gained momentum. Meta-analytic evidence suggests biologics, including MSCs, may provide longer-lasting improvements in pain and function (17). Umbilical cord-derived MSCs have demonstrated clinical and regenerative potential in human knee OA, when compared with other MSC sources and HA controls (18, 19). In veterinary medicine, eCB-MSCs have shown both safety and potential to slow OA progression and improve the joint environment (20), across traumatic models and naturally occurring OA (21–23).

When using allogeneic MSCs, products may be generated from a single donor or from pooled donors. While clinical outcomes may be comparable, pooling can reduce donor-to-donor variability and improve product standardization, including more consistent cell yield and potentially less variability in potency (24, 25). In clinical practice, MSCs are also frequently used in combination therapies to maximize synergistic effects, for example co-administration with HA (26). In this context, HA is proposed to provide more than additive analgesia, potentially supporting MSC retention and/or localization within the injured joint and promoting chondrogenic responses (27, 28).

Currently, clinical OA diagnosis typically begins with a lameness workup to localize the affected joint, followed by radiographic evaluation. However, radiography has limited sensitivity for detecting early OA changes (29). Because early recognition may influence both prognosis and treatment decision, MRI has been increasingly adopted in both human and equine research and clinical applications, to characterize OA progression and evaluate therapeutic responses (30–38). The use of MRI-based scoring approaches improves consistency and reproducibility of structural outcome assessment across studies.

The aim of this prospective randomized experimental study was to evaluate the safety and efficacy of pooled eCB-MSCs in HA for the treatment of surgically induced OA in the fetlock joint of horses, compared to a saline control group. In addition, synovial fluid biomarkers were measured to assess joint inflammation. We hypothesize that pooled cryopreserved non-activated eCB-MSCs+HA are safe and effective in reducing joint pain and inflammation in an injury-induced equine OA model when compared to the saline treatment.

## 2 Materials and methods

### 2.1 Animals

A total of 12 Standardbred mares retired from racing and owned by the University of Guelph were enrolled over a 2-year period. The mean age was 10.5 ± 5.5 years (range, 2–17 years). All horses underwent clinical examination by an ACVS diplomate and an ACVSMR resident to confirm eligibility for inclusion. Only horses with an AAEP Lameness Scale of 1/5 or less were considered. All 12 horses completed the study and remained enrolled until week 13.

Neither a formal *a priori* sample size nor power calculation were performed. The group size of six horses per cohort was constrained by housing capacity, the intensive daily management and treadmill conditioning required, shared access to imaging and surgical facilities, and funding, consistent with large-animal experimental studies.

The project received approval from the University of Guelph Animal Care Committee (ACC) under the Animal Utilization Protocol number 4616, in compliance with Ontario regulations and the Canadian Council on Animal Care (CCAC).

### 2.2 Study overview

One week after arrival, all horses underwent an initial assessment that included both objective (Lameness Locator®) and subjective lameness evaluations to confirm soundness and rule out significant pre-existing OA. Ultrasonographic and radiographic examinations of the metacarpophalangeal joints were also performed to identify any lesions that could interfere with the study. If mild OA was identified in one fetlock, that joint was selected for surgery. An equal number of right and left MCP (metacarpophalangeal) joints (*n* = 6 each) were selected. During this same week, all horses were acclimated to treadmill exercise.

The first arthroscopic surgery was performed to create an OC fragment in the selected MCP joint. Horses were randomly assigned into two groups (*n* = 6 per group). At 6 weeks (time-of-treatment), the intervention group received intra-articular injections consisting of 1 mL containing 10 million eCB-MSCs mixed at injection time with 2 mL of HA (HY-50® 17 mg/mL, Dechra Veterinary Products, Canada), into the affected MCP joint, while the saline group received an intra-articular injection of 3 mL of 0.9% sterile Sodium Chloride (Surgo surgical supply, Canada). At week 12 (post-surgery), a second surgery was performed to remove the fragment and collect synovial membrane and cartilage samples from the distal metacarpal condyle and the proximal first phalanx. These samples were processed for histologic and immunohistochemical analysis. Videos of the articular cartilage were recorded and independently evaluated by a board-certified surgeon using a modified scoring system, as described below. Based on prior studies of OC lesion development, the 13-week time point was selected as the endpoint (11).

Throughout the study period, horses underwent regular physical examinations, subjective (video-recorded), and objective (Lameness Locator®) lameness evaluations. Joint circumference and joint temperature were routinely recorded using an infrared thermometer (Maximum Thermal Imager, CTC, Canada). Ultrasonographic assessments were performed every 4 weeks to monitor for the development of osteophytes, synovitis, or joint effusion.

Radiographic evaluations were conducted before surgery (pre-surgery), and 6 weeks after treatment (post-treatment). Two MRI scans were performed at weeks 5 and 11 (post-treatment). Radiographs and MRIs were reviewed and graded by a diplomate of the American College of Veterinary Radiology and a resident of the American College of Veterinary Sports Medicine and Rehabilitation.

Synovial fluid was collected for cytologic and molecular biomarker analysis at the following time points: pre-surgery, prior to the intra-articular injection (time-of-treatment) and at 13 weeks (post-treatment). On synovial sampling days, horses received one dose of phenylbutazone (2 mg/kg) and 150 mg of xylazine to facilitate the procedure.

Twenty-three cytokines were quantified using the MILLIPLEX® Equine Cytokine/Chemokine Magnetic Bead Immunoassay (Sigma-Aldrich, St. Louis, MO, United States), and PGE2 concentrations were measured using a competitive enzyme immunoassay kit (R&D Systems, Minneapolis, MN, United States).

**Figure 1:**
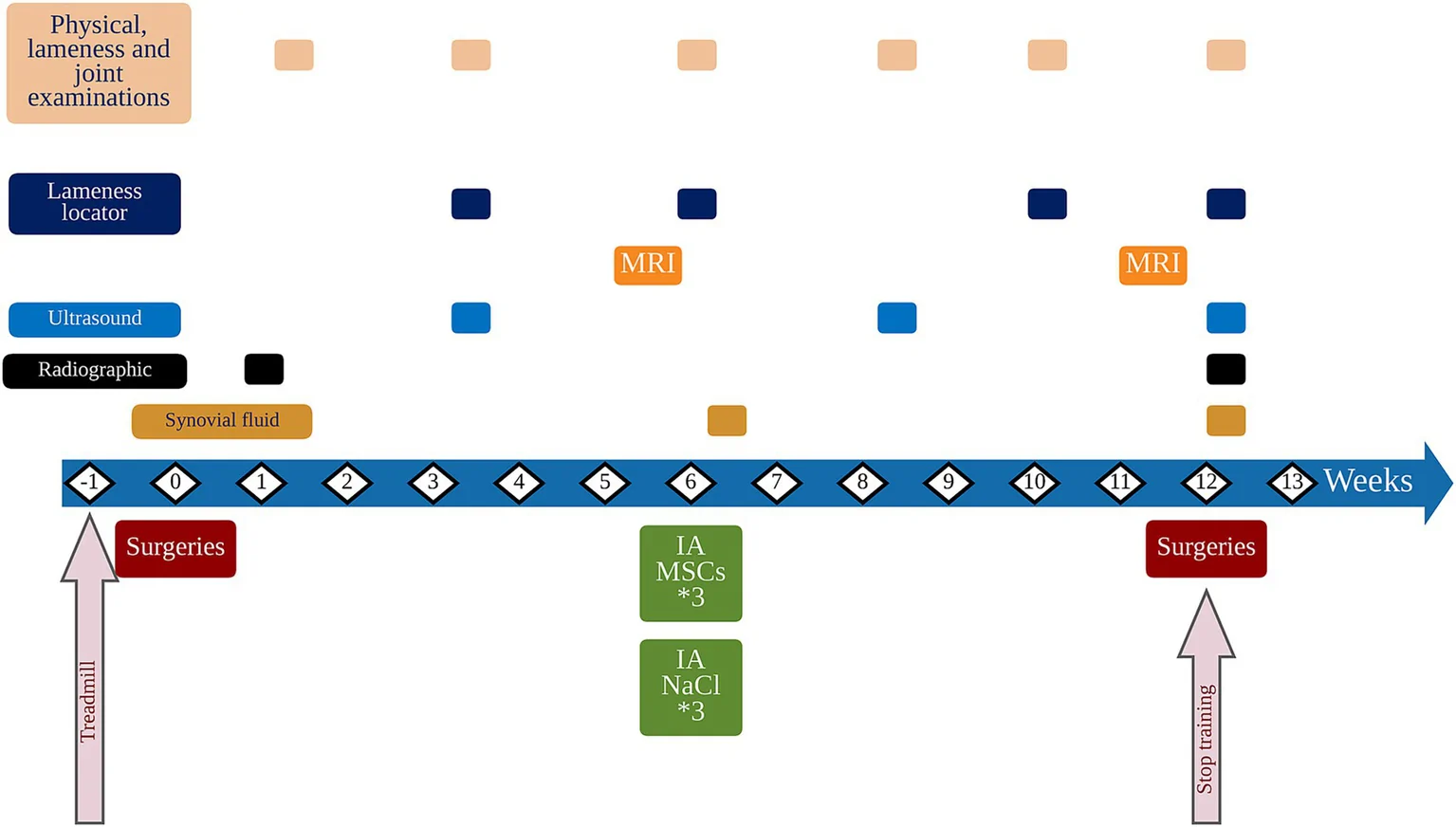
Timeline of the study design. Prior to surgery, all horses completed a one-week treadmill acclimation period. An osteochondral fragment model was then surgically induced, after which horses were randomly allocated to receive an intra-articular injection of eCB-MSC + HA (*n* = 6) or saline as control (*n* = 6). The study was conducted over two consecutive years, with six horses per cohort. Clinical evaluations, diagnostic imaging and synovial fluid sampling were performed throughout the study.

A summary of study events is presented in the timeline (Figure 1).

### 2.3 Surgery

Arthroscopic surgeries were performed on study days 0 and 1 under general anesthesia, with three horses operated on each day, by board-certified surgeons. Horses were anesthetized and positioned in dorsal recumbency. Joint selection was based on the presence of mild, identifiable pathology that, although not sufficient to cause lameness or warrant exclusion from the study, could potentially contribute to OA progression. Three right and three left fetlocks were selected per study group.

The surgical technique was based on previously described models by Boyce et al. (11) and Broeckx et al. (12), with minimal modifications. The dorsal aspect of the selected MCP joint was arthroscopically evaluated through a routine dorsolateral portal. The joint was inspected for pre-existing lesions, and the arthroscopic appearance was video recorded for later assessment.

An OC fragment was created through a proximomedial portal using a standard straight 8 mm osteotome tapped with a mallet. The osteotome was placed proximal to the dorsomedial edge of the proximal phalanx. A custom-made template at 55 degrees was used to approximate the insertion angle, but variations in the proximal phalanx contour required minor subjective adjustments for accurate positioning. The fragment was left attached to the dorsal joint capsule.

**Figure 2:**
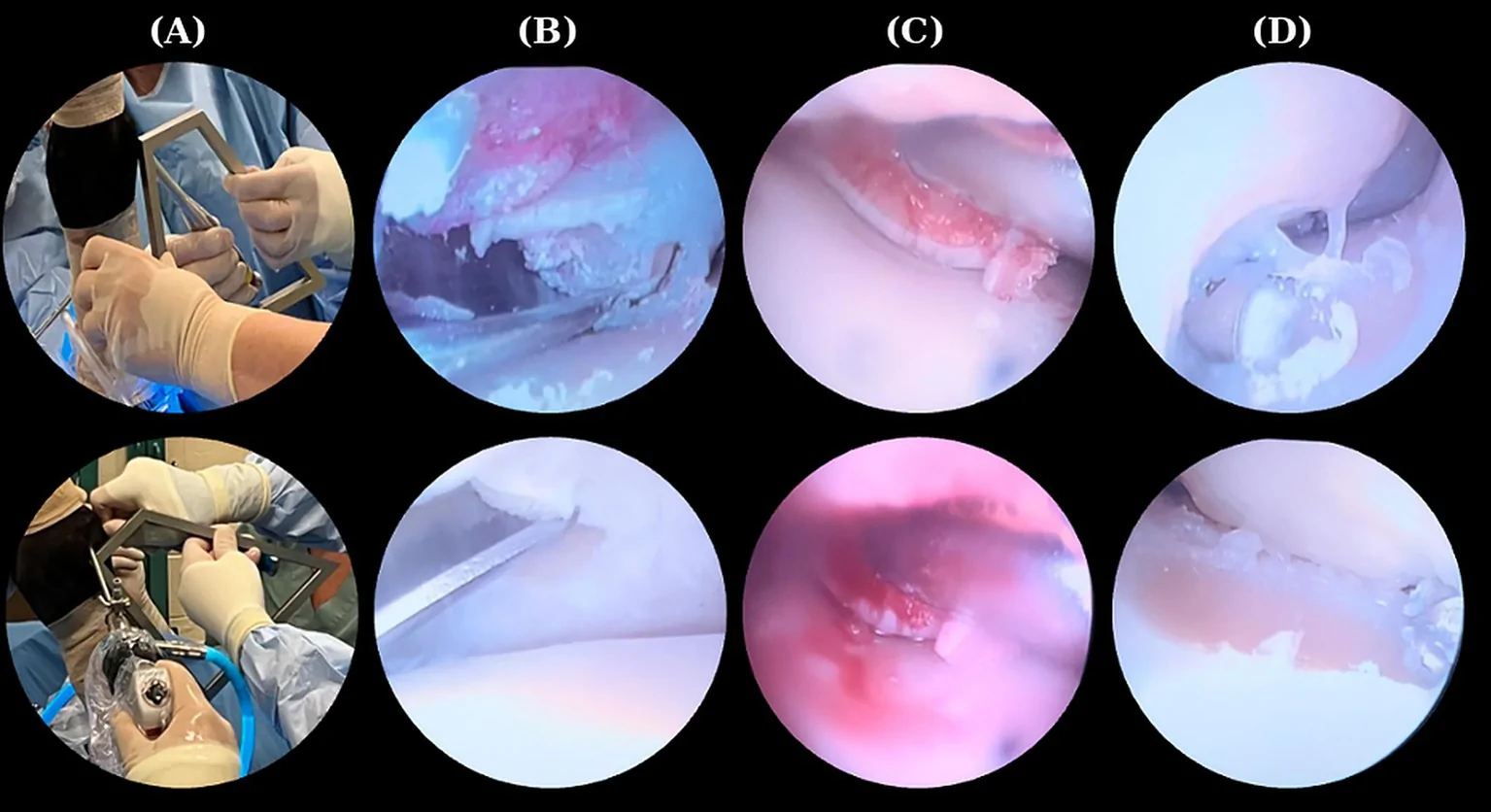
Surgery technique used for the osteochondral fragment and groove creation. Column **(A)**: custom made template wedge to standardize creation of osteochondral fragments, measuring 180 mm (length), with a 55-degree angle with respect to the dorsal aspect of the third metacarpal bone. Column **(B)**: 8 mm osteotome creating a fragment on the dorso-medial aspect of the proximal phalanx. Column **(C)**: arthroburr osteochondral fragment bed debridement. Column **(D)**: arthroburr horizontal groove creation on the dorsal aspect of the medial condyle.

At this point, lactated Ringer’s solution was replaced with CO₂ to distend the joint prior to debridement, ensuring that cartilage and bone debris remained within the joint to maximize synovial irritation and inflammatory response. An arthroburr was then used to debride the subchondral bone bed and create a horizontal cartilage groove on the dorsal aspect of the medial condyle of the third metacarpal bone (Figure 2). The skin was routinely closed, and postoperative care included stall rest, bandaging, follow-up, and suture removal.

Twelve weeks after the initial surgery, a second arthroscopy was performed and digitally recorded. Synovial membrane samples were collected from the dorsomedial aspect of the joint, and cartilage samples were obtained from both the fragment bed and the opposing surface on the medial metacarpal condyle. These samples were sent in buffered formalin to the Animal Health Laboratory of the University of Guelph, for a histopathology analysis by a board-certified pathologist. Post-operative care was the same as for the first surgery.

All horses were premedicated prior to both surgeries with procaine penicillin G (20,000 IU/kg) and phenylbutazone (4 mg/kg). Immediately after the first surgery, the operated joints were injected with 120 mg of mepivacaine hydrochloride. That evening, 45 mg of morphine was administered intramuscularly, along with a repeat dose of phenylbutazone at the same dosage.

For the second surgery, horses received the same premedication protocol. Post-operatively, 45 mg of morphine was injected intra-articularly, and phenylbutazone was administered as needed for analgesia over the following days.

### 2.4 Equine umbilical cord blood collection

The Animal Care Committee at the University of Guelph has previously approved the collection protocol by the AUP 06R076. Umbilical cord venous blood was collected from foals immediately after foaling using a 16-gauge hypodermic needle connected to a 450 mL blood transfusion collection bag (Fenwal) containing CPDA as an anticoagulant. Samples were stored and transported at ambient temperature to the laboratory for processing.

### 2.5 eCB-MSC isolation and expansion

MSCs were harvested from blood of the umbilical vein of foals from Thoroughbred or Standardbred donor broodmares of various ages only if they were healthy at the time of foaling and had normal complete blood counts and a negative Coggins test. The NC fraction was isolated from cord blood using RBC lysis as previously described 39. The cells were transferred to a 1-layer Corning CellStack culture chamber and incubated at 38 °C in a humidified atmosphere containing 5% CO₂. Media changes were performed every 1–3 days until colonies of spindle-shaped fibroblast-like cells, characteristic of MSCs, were observed. Cultures were monitored daily for contamination and colony formation. When cell confluence reached 60–80%, sub-culturing was performed. Cells were detached and seeded in Corning CellStack chambers. Cells were expanded for 3 passages and frozen into preliminary cell banks. Cells were submitted to the Animal Health Laboratory (University of Guelph) for antigen PCR testing and were negative for Equine Pegivirus, Equine Parvovirus, Equine Hepacivirus, Theiler’s Disease Associated Virus, West Nile Virus, Equine Eastern Encephalitis Virus, Equine Herpesvirus-1, and Equine Viral Arteritis Virus.

To qualify MSCs for clinical use, cryopreserved MSCs from each donor were thawed, expanded to passage 5, and characterized by flow cytometry for expression of CD29, CD44, CD90, CD105, CD146, CD34, CD45, MHC I, and MHC II. Immunomodulatory potency was assessed using a CFSE-based mixed lymphocyte reaction (MLR) assay as previously described (39). Briefly, CFSE-labelled equine PBMCs were stimulated with concanavalin A and co-cultured with MSCs for 5 days. PBMC proliferation was quantified by flow cytometry based on CFSE dilution and compared with stimulated PBMC controls to confirm the ability of MSCs to suppress lymphocyte proliferation.

Only MSC cultures meeting the predefined immunophenotypic profile (positive for CD29, CD44, CD90, CD105, CD146, and MHC I, with minimal expression of CD34, CD45, and MHC II), demonstrating suppression of stimulated PBMC proliferation, and testing negative for the above pathogens were qualified for clinical cell expansion. Cryopreserved passage 3 MSCs from five qualified donors were subsequently thawed, and equal numbers of viable cells from each donor were combined to generate a pooled cell population. The pooled MSCs were expanded to passage 5, cryopreserved and used for clinical application.

### 2.6 Cryopreservation and thawing of eCB-MSCs

At the time of cryopreservation, cell culture samples were tested for sterility by bacterial culture and endotoxin testing. Cells were detached with TrypLE Express (1 mL per 10–12 cm^2^ of culture surface), resuspended in expansion medium, and centrifuged at 400 × g for 5 min. The supernatant was discarded, and cells were resuspended at a concentration of 20 million cells/ml. Cold cryomedium (low-glucose DMEM with 20% DMSO) was added at a 1:1 ratio for a final concentration of 10 million cells/ml. The aliquots were transferred to CellSeal cryogenic vials (SEXTON Biotechnologies), stored overnight at −80 °C in a CellSeal freezing container (SEXTON Biotechnologies), and then transferred to liquid nitrogen storage (Thermo Fisher).

For clinical applications, cryovials were thawed using the CellSeal Automated Thawing System (SEXTON Biotechnologies) for 5 min prior to injection. In accordance with the experimental protocol, a 1 mL cell suspension containing 10 million cells (average cell viability of 80.1% ± 5.9) was prepared for injection.

### 2.7 Treatment rationale

The 10 million eCB-MSC dose was selected based on prior efficacy and dose-ranging data (22, 40). A clinical pilot study in horses with metacarpophalangeal joint OA reported efficacy of allogeneic umbilical cord-derived MSCs at this dose (22), and a subsequent clinical synovitis trial by our group comparing 10 vs. 20 million eCB-MSCs combined with HA demonstrated comparable safety and clinical outcomes at both doses (40). The lower effective dose was therefore selected for this experimental study. HA was co-administered as the combination most relevant to clinical use, for the reasons previously outlined. As the study did not include eCB-MSC-alone and HA-alone arms, their individual contributions could not be distinguished.

### 2.8 Exercise protocol

All horses arrived at the Equine Sports Medicine and Rehabilitation Centre (ESMRC) 1 week prior to the initial surgery (week 0). Horses remained barefoot throughout the study and received regular farrier trimming. During the acclimation week, horses were gradually introduced to walk and trot on the treadmill.

Two days after surgery, horses were hand-grazed with restricted movement. By day 4 post-surgery, they were turned out in small 8 × 8-meter grass paddocks to allow controlled movement. One week after surgery, horses were hand-walked for 15 min twice daily. By day 10, treadmill training initiated, beginning with a 12-min walk followed by three intervals of 1-min trot and 1-min walk. Two days later, the trotting intervals were increased to 2 min.

Horses were progressively conditioned to complete the full treadmill protocol, conducted once daily, 5 days per week. The final protocol consisted of 2 min of walking at 7 km/h, followed by 5 min of trotting at 15 km/h, then 1 min of walking at 7 km/h, followed by another 5 min of trotting at 15 km/h, and finally a 2-min walk at 7 km/h. This protocol was adapted with minimal modifications from a previously described regimen (12) to maintain zone 2 exercise during the trotting phases (41).

After arthrocentesis, horses were rested the following day and resumed treadmill work on the second day.

### 2.9 Clinical assessment of the joint

All the clinical evaluations were performed by the same ACVSMR resident, who was blinded to the treatments. The circumferences of the operated MCP joints were consistently measured at the level of the most prominent aspect of the proximal and palmar recess of the joint, just proximal to the metacarpal condyles. Similarly, temperature was measured in this recess using a laser thermometer, allowing for multiple readings to obtain a consistent value. Additionally, joint effusion was graded from 0 (normal) to 4 (severe), adapted from a previously reported scoring system (42). Edema, when present, was scored and added to the same joint effusion grading.

The subjective degree of lameness was graded using the AAEP scale (43), modified to include 0.5 grades between 0 and 1, as previously described (40); (Supplementary Table S1). At the time of the examinations, the same clinician evaluated the degree of lameness, and a recorded video also allowed a remote evaluation by a second clinician. To complement the subjective lameness exam and allow minor gait changes to be detected, an objective lameness examination using the inertial sensor system Lameness Locator® (Equinosis LLC, United States) was performed, using the computed vector sum (measured in mm) from the comparison of the maximum and minimum head height differences (44).

During the final third of the study, a passive flexion test (45–60 s of passive flexion, depending on each horse’s tolerance to the manipulation) was included in the lameness evaluations to differentiate and exacerbate the lameness, particularly since all horses were gradually improving clinically, making distinctions among them more complex. By that point, some horses in this study were only 1/5 lame on a straight line; therefore, after the flexion tests, lameness increased significantly, and in these cases, the trot following the flexion test was graded using the same scale for analysis purposes.

### 2.10 Ultrasonographic assessment

A 7.5 MHz linear transducer (Logiq e, GE Healthcare, United States) was used to obtain longitudinal and transverse images from the fetlock joints. A systematic assessment was performed by the same clinician (RML), on the palmar and dorsal recesses to be able to grade the joints based a previously reported scoring system (42). Additionally, the entire dorsal aspect of the joint was evaluated longitudinally, focusing on early osteophyte formation and changes related to the OC fragment.

The OA osteophyte scoring was not included because of inconsistent acquisition of longitudinal images from the dorsal aspect of the joint at the start of the study. Therefore, a total ultrasound OA score was not provided in this project; however, the images obtained at the end of the project served as a comparison of OA progression in the later stages across different imaging modalities.

### 2.11 Radiographic assessment

Bilateral metacarpophalangeal joint radiographs were taken before the surgical procedures with a digital radiographic system (Canon 801, 11*14 cassette with a 90 kV/20 mA generator); the views included a dorsopalmar, dorsolateral-palmaromedial oblique, a dorsomedial-palmarolateral oblique, a lateromedial and a flexed lateromedial. One week after the surgeries, only dorsolateral-palmaromedial oblique views were taken to identify the OC site. The following sets of radiographs were unilateral and included all the previous views.

The MCP joint was divided in 13 sites for the osteophyte radiographic scoring (Supplementary Figure S1); dorsal (1), palmar (2), lateral (3) and medial (4) aspects of the third metacarpal (MC3) condyles, the dorsal (5), dorso-lateral (6), dorso-medial (7), palmaro-lateral (8), and palmaro-medial (9) P1, proximal (10) and distal (11) aspects of the lateral proximal sesamoid bone (PSB) and the proximal (12) and distal (13) aspects of the medial PSB.

Radiographic osteophytes were blindly graded by an ACVR diplomate and a ACVSMR resident based on severity, using a grading scale from 0 (normal) to 3 (severe), following criteria established in this OA study and other fetlock OA models (13, 23, 29). (Supplementary Figure S2 provides an illustration of comparative osteophyte findings between groups). Sclerotic changes were not included in the scoring due to previously reported variability and limited reliability (29). A total of 13 sites were assessed per joint, yielding a maximum possible score of 39.

### 2.12 MRI scoring system

A 1.5 T scanner (Signa Excite II; General Electric Medical Systems) was used with the horses under general anesthesia. The images were obtained at weeks 5 and 11 following OA creation, before and after the first treatment. The sequences used are displayed in Supplementary Table S2.

Eight regions used for the WORMS scoring system were defined in the MCP joint (Supplementary Figure S1) as follows: lateral and medial condyles (I–II), sagittal ridge (III), lateral and medial proximal phalangeal joint (P1) (IV–V), sagittal groove (VI), and lateral and medial sesamoids (VII–VIII) (38).

MRI images were blindly graded by an ACVR diplomate and a ACVSMR resident using a modified WORMS system previously proposed for the equine MCP joint (Supplementary Table S3) (38). High and low signal bone lesions were quantified as percentages of the affected area, measured from at least two imaging planes. All remaining parameters were scored based on established grading systems (29, 37, 38). Synovial membrane thickness on the dorsal aspect of the joint was scored separately using a system described by Smith et al. (37) as follows: 0 = normal, 1 = mild, 2 = moderate, 3 = marked or severe. Joint effusion was similarly graded: 0 = normal, 1 = mild, 2 = moderate, 3 = marked or severe (29). The maximum possible WORMS score was 158.

To account for individual variation, the MRI of the contralateral (non-operated) limb served as a comparison baseline for each horse. Synovial thickness was measured on a sagittal view at the dorsal aspect of the joint. For the operated limb, thickness measurements equal to or below that of the contralateral limb were graded as 0. An increase of ≤1 mm was considered grade 1 (mild synovitis), 1–2 mm increase as grade 2 (moderate synovitis), and >2 mm increase as grade 3 (marked or severe synovitis).

### 2.13 Arthroscopic scoring

Arthroscopic photographs and/or videos were reviewed for all horses during both the initial arthroscopic surgery (prior to fragment creation) and the second-look arthroscopy at the time of fragment removal.

An arthroscopic scoring system was employed, adapted from previous studies (11, 45, 46), to specifically assess injury associated with OC fragmentation and modified to reflect the surgical technique used in this study, which included burr creation of a groove in the MC3 bone, graded from 0–4. A total of 13 categories were graded by a blinded ACVS diplomate, and scores were summed to yield a total arthroscopic score ranging from 0 to 43.

### 2.14 Analysis of synovial fluid

Synovial fluid was collected in EDTA tubes, and 0.5 mL was taken to the Animal Health Laboratory of the University of Guelph, for routine analysis including the total nucleated cell counts (TNCC) (Coulter Counter, Beckman Coulter, United States), total protein concentration by refractometry, and microscopic smear analysis by a board-certified clinical pathologist. The remainder of the synovial fluid was immediately taken to the laboratory to be processed and stored as follows: EDTA tubes were centrifuged at 4,000 x *g* for 5 min (Thermo Scientific Legend X1 centrifuge); the supernatant was transferred into 0.5 mL microcentrifuge tubes (Eppendorf ®; Hamburg, Germany) in 250 μL volumes. All samples were snap frozen in liquid nitrogen and stored at −80 °C until further cytokine and chemokine quantification.

23 cytokines and chemokines were analyzed with the fluorescent MILLIPLEX® Equine Cytokine/Chemokine Panel (MilliporeSigma, Burlington, MA, United States) (47). After thawing the samples on ice, 100 μL of synovial fluid were hyaluronidase-digested with 0.5 IU of bovine testicular hyaluronidase (100 IU hyaluronidase/ml acetate buffer), (H3506, Sigma-Aldrich, St. Louis, MO, United States) (48), at 37 °C for 30 min, collecting the supernatant after a centrifugation at 12,000 x *g* for 10 min at 4 °C, based on methods from a previous study (49). Then, the Milliplex® manufacturer’s protocol was followed (50). Briefly, synovial fluid was aliquoted into each of 96 wells, the plates were sealed and stored for 18 h at 4 °C, and then placed on a shaker at room temperature for 10 min before reading using a Bio-Plex® 200 Multiplex Immunoassay system (Bio-Rad, Mississauga, Canada).

For the analysis of PGE2, the manufacturer’s suggested protocol was followed using the Prostaglandin E2 Parameter Assay Kit (Bio-Techne, Minneapolis, MN, United States), with thawed synovial fluid incubated on ice and read in a microplate reader (Synergy HT, Gen 5 BioTek software, Vermont, United States).

### 2.15 Statistical methods

Statistical analyses were performed using SAS version 9.4 (SAS Institute Inc., Cary, NC, United States). All tests were two-sided, with statistical significance set at *p* < 0.05.

Continuous outcomes were analyzed using linear mixed-effects models (LMM) fitted by restricted maximum likelihood. Least-squares means (estimated marginal means) with 95% confidence intervals were reported, and prespecified contrasts between treatment groups were evaluated using model-based t tests; overall fixed effects were assessed using F tests. Model assumptions were evaluated using residual diagnostics, including visual inspection of residual plots and normality tests (Shapiro–Wilk and Anderson–Darling). When heteroscedasticity was indicated, alternative variance structures were compared using Akaike’s information criterion, and the best-fitting structure was retained. For the statistical analysis, baseline was considered at week 5 (time-of-treatment).

For synovial fluid cytokine values, the LMM included horse ID included as a random effect to account for the repeated or clustered observations. Fixed effects included treatment, fragment size, baseline cytokine values, and the interactions treatment-by-fragment size and treatment-by-baseline cytokine values. Non-significant fixed effects were removed stepwise to simplify the model (removal criterion *p* > 0.15). When interactions with baseline cytokine values or fragment size were present, treatment comparisons were estimated at prespecified baseline values along the slope.

For joint circumference, the LMM included treatment group, baseline circumference, and fragment size as fixed effects; adjusted group means (LS-means) with 95% CIs were estimated and group differences were derived from these LS-means.

For the objective Lameness Locator measure, an LMM was fitted with the objective lameness outcome as the dependent variable, treatment group as a fixed effect, and baseline objective lameness as a covariate, including a treatment-by-baseline interaction; treatment effects were summarised using LS-means with 95% CIs.

WORMS and subjective lameness scores were analysed using nonparametric methods. Within-group change from baseline was assessed using the Wilcoxon signed-rank test, including paired comparisons of baseline versus follow-up for radiographic scores (pre-surgery vs. discharge), WORMS (first vs. second MRI), and arthroscopy scores (first surgery vs. second surgery). For subjective lameness, the change in score from time-of-treatment to post-treatment (week 13) was compared between groups using a two-sided Mann–Whitney *U* test with a baseline-adjusted analysis as confirmation of the between-group treatment effect.

Correlations between WORMS scores and synovial fluid cytokine values were assessed using Pearson’s product–moment correlation. For each cytokine, the Pearson correlation coefficient (r) was calculated with WORMS, and a two-sided test of the null hypothesis of zero correlation was performed.

The relationship between the objective Lameness Locator measure and the subjective lameness score at each time point was evaluated using Spearman’s rank correlation coefficient, with corresponding two-sided *p*-values for the null hypothesis of zero correlation. The relationships among WORMS scores, radiographic scores, and arthroscopic scores were also assessed using Spearman’s rank correlation, with corresponding two-sided p-values for the null hypothesis of zero correlation.

## 3 Results

### 3.1 Lameness outcomes

**Figure 3:**
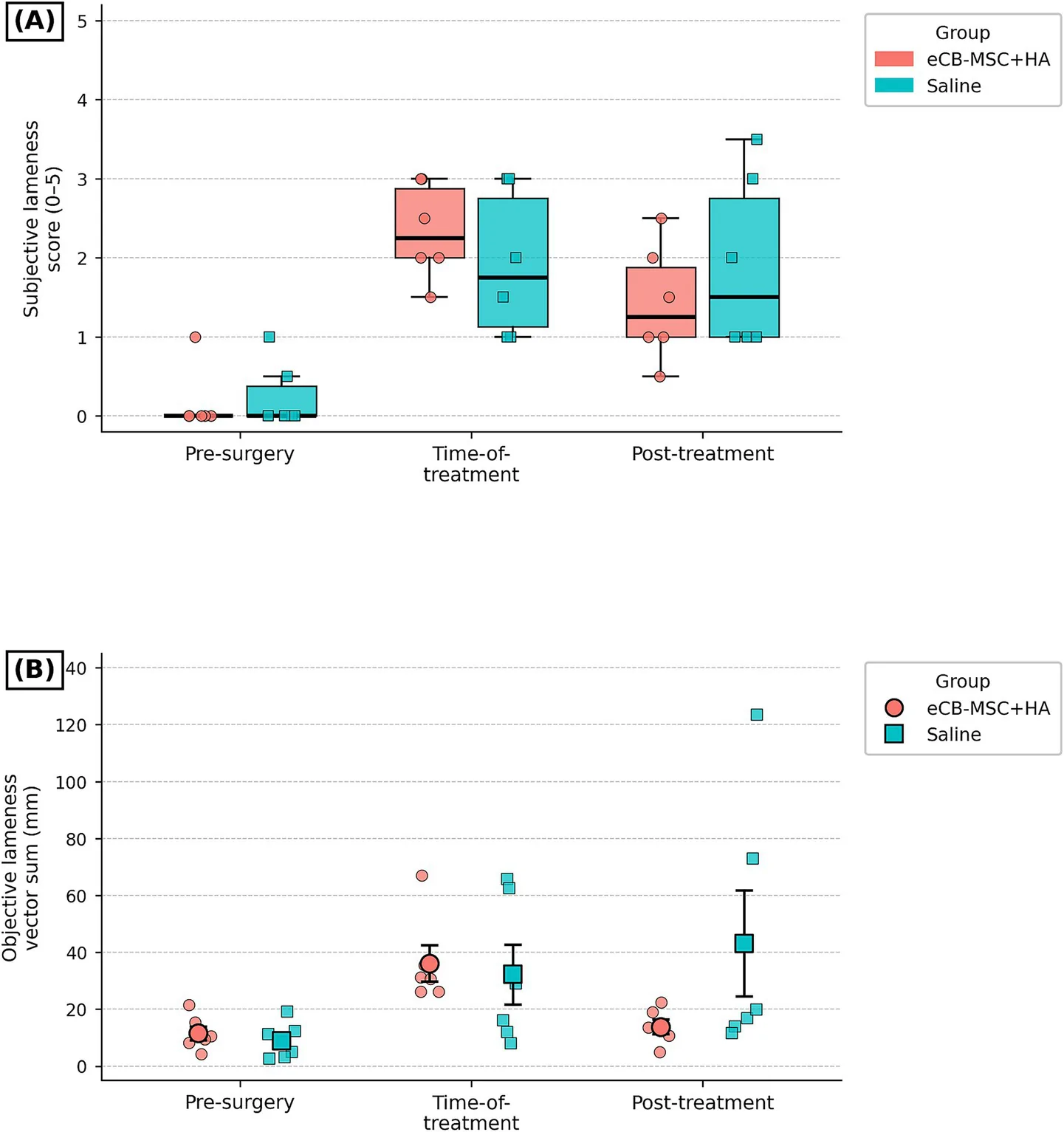
Lameness assessments across study timepoints. **(A)** Subjective lameness scores (0–5 scale) shown as box plots (median, interquartile range; individual values as points) and **(B)** objective lameness vector sum (mm) shown as individual values with group mean ± SEM, at weeks 0 (pre-surgery), 5 (time-of-treatment), and 13 (post-treatment) for horses receiving eCB-MSC + HA (*n* = 6) or Saline (*n* = 6). The change in subjective lameness from week 5 to week 13 differed significantly between groups (*p* = 0.035). For objective lameness, a significant treatment-by-baseline interaction was detected (*p* = 0.016); adjusted post-treatment LS-means ± SE at week 13 were significantly lower in the eCB-MSC + HA group compared to Saline (14.0 ± 6.7 mm vs. 46.3 ± 6.7 mm; *p* = 0.009).

Results for both scoring systems are presented in Figure 3. Objective lameness post-treatment showed evidence of a treatment-by-baseline interaction (*F* = 9.38, *p* = 0.016). In the saline group, post-treatment lameness increased by 1.60 ± 0.28 mm for each 1 mm higher lameness at time-of-treatment, whereas in the eCB-MSC + HA group the slope was −0.08 ± 0.61 mm (slope difference 1.68, *p* = 0.015). The adjusted post-treatment LS-mean was 14.0 ± 6.7 mm (95% CI –1.49 to 29.51) in the eCB-MSC + HA group and 46.26 ± 6.7 mm (95% CI 30.85 to 61.68) in the saline group (adjusted between-group difference, −32.3 ± 9.5 mm, 95% CI –54.1 to −10.4; *p* = 0.009).

Subjective lameness scores decreased from time-of-treatment to post-treatment. The reduction was greater in the eCB-MSC + HA group (median change from 2.25 to 1.25) than in the saline group (median change from 1.75 to 1.50); the between group difference in change was significant (exact Wilcoxon rank-sum, two-sided *p* = 0.035), and remained so after adjusting for baseline score (baseline-adjusted permutation analysis, *p* = 0.037).

At time-of-treatment there was a strong correlation between both lameness scoring systems (*r* = 0.899. *p* = <0.001) which remained significant post-treatment (*r* = 0.657, *p* = 0.020).

### 3.2 Clinical joint assessments

Median effusion scores did not differ at time-of-treatment or post-treatment, with no evidence of within-group change (*p* = 0.773) or between-group differences (*p* = 0.487). In contrast, after adjustment for time-of-treatment circumference and fragment size, post-treatment joint circumference was higher in the eCB-MSC + HA group than in the saline group (adjusted mean difference 0.85 cm, SE 0.31, *p* = 0.024), with adjusted means of 24.63 cm (SE 0.21) in the eCB-MSC + HA group and 23.78 cm (SE 0.21) in the saline group.

### 3.3 Diagnostic imaging and arthroscopic scores

WORMS outcomes differed by group over time. In the eCB-MSC + HA group, the median change from time-of-treatment to post-treatment was −1.0 (IQR − 3 to 0; *p* = 0.312). In the saline group, the median change was +2.5 (IQR + 2 to +4; *p* = 0.030). Change scores differed between groups (Wilcoxon rank-sum test *p* = 0.020). Synovial thickness, assessed as part of the WORMS framework, was summarised descriptively: mean synovial thickness decreased from 7.1 ± 1.6 mm time-of-treatment to 5.3 ± 1.0 mm post-treatment in the eCB-MSC + HA group (mean change −1.7 mm) and from 6.9 ± 1.1 mm to 5.8 ± 0.9 mm in the saline group (mean change −1.1 mm).

The mean OC fragment size measured with MRI was 26.1 mm^2^ (± 19.9), the median was 18.3 mm^2^ (IQR 27.05), and the range was 6.9 mm^2^ to 65 mm^2^. During the first MRI, one horse with the largest measured OC fragment (65 mm^2^) also showed an approximately 1 cm unicortical parasagittal fracture in the proximal phalanx, aligned with the OC fragment bed (Supplementary Figure S3).

Radiographic scores were obtained for all horses pre-surgery and compared with post-treatment scores. Pre-surgery scores were low in both groups (median 0; eCB-MSC + HA group range 0–1, saline group range 0–7). Post-treatment, median scores increased to 2.5 (1–8) in the eCB-MSC + HA group and 8 [0–10] in the saline group, with no significant difference between groups (*p* = 0.57).

Arthroscopic scores increased from surgery 1 (pre-fragment creation) to the week 12 arthroscopic assessment (post-treatment) in both groups. Median scores increased from 0.5 (range 0–9) to 12.5 (range 10–21) in the eCB-MSC + HA group and from 2 (range 0–6) to 21 (range 10–23) in the saline group: both increases were statistically significant (Wilcoxon signed-rank test, *p* = 0.031 for both groups). The median change from pre- to post-treatment was +11.5 points (IQR + 11.0 to +12.8) in the eCB-MSC + HA group and +15.5 points (IQR + 13.5 to +19.8) in the saline group, with no significant between-group difference (Wilcoxon rank-sum test, *p* = 0.108).

**Figure 4:**
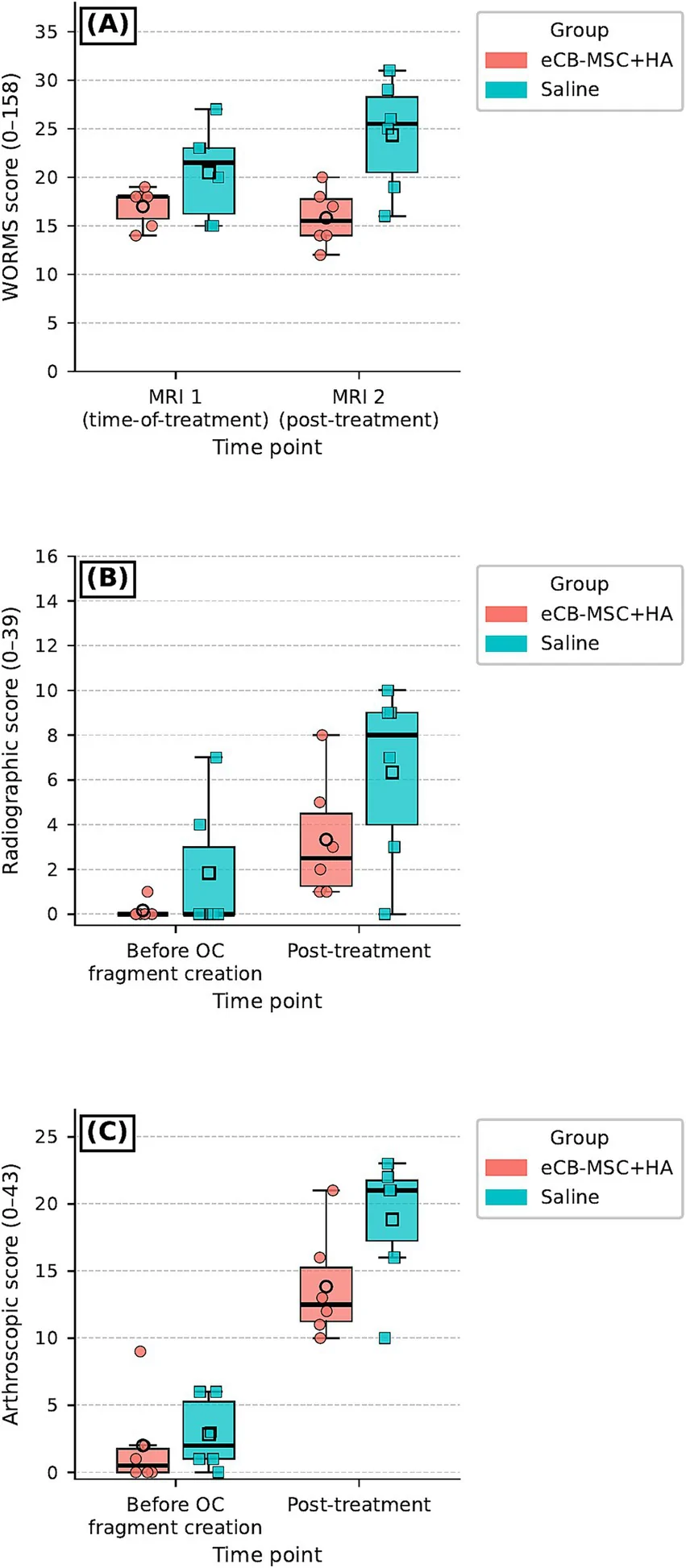
Joint scoring outcomes across study timepoints. **(A)** MRI-based WORMS scores (0–158) at MRI 1 (time-of-treatment) and MRI 2 (post-treatment). **(B)** Radiographic scores (0–39) and **(C)** arthroscopic scores (0–43), assessed before osteochondral (OC) fragment creation (pre-surgery) and post-treatment, in horses receiving eCB-MSC + HA (*n* = 6) or Saline (*n* = 6). Data are presented as box plots (median, interquartile range, and whiskers 1.5 × IQR); open symbols indicate group means; individual values are shown as points. WORMS scores change from MRI 1 to MRI 2 differed significantly between groups (Wilcoxon rank-sum test, *p* = 0.020), with the Saline group increasing (median +2.5, IQR + 2 to +4; *p* = 0.030) and the eCB-MSC + HA group remaining unchanged (median −1.0, IQR − 3 to 0; *p* = 0.312). Radiographic and arthroscopic scores increased in both groups post-treatment; no significant between-group differences were detected (*p* = 0.57 and *p* = 0.108, respectively).

WORMS correlated strongly with both radiographic scores (*r* = 0.831, *p* < 0.001) and arthroscopic scores (*r* = 0.811, *p* = 0.001). Radiographic and arthroscopic scores were also highly correlated (*r* = 0.851, *p* < 0.001). Figure 4 describes the scores comparison between groups.

### 3.4 Synovial fluid total nucleated cell counts and total protein

There were no statistically significant effects of treatment, fragment, or time-of-treatment TNCC on either outcome. Total protein did not differ between the MSC and saline groups (*p* = 0.448); the estimated marginal mean was 19.1 (SE 0.655) in the MSC group and 19.9 (SE 0.655) in the saline group. TNCC also did not differ between groups (*p* = 0.670); the estimated marginal mean was 0.629 (SE 0.151) in the MSC group and 0.528 (SE 0.151) in the saline group.

### 3.5 Markers without evidence of treatment-related differences

There was no evidence of a treatment effect on synovial fluid MFI levels for Eotaxin (*p* = 0.702), fibroblast growth factor 2 (FGF-2) (*p* = 0.685), granulocyte colony-stimulating factor (GCSF) (*p* = 0.596), growth-regulated oncogene (GRO) (*p* = 0.932), interferon-gamma (IFN-γ) (*p* = 0.200), interferon-gamma-induced protein 10 (IP-10) (*p* = 0.391), interleukin 1 alpha (IL1*α*) (*p* = 0.186), interleukin 6 (IL-6) (*p* = 0.110), interleukin 10 (IL-10) (*p* = 0.550), interleukin 12 (IL-12) (*p* = 0.762), and monocyte chemoattractant protein 1 (MCP-1) (*p* = 0.743), or on prostaglandin E2 (PGE2) concentration (*p* = 0.540). RANTES was not formally analyzed because too few values were available for reliable statistical testing.

### 3.6 Markers with evidence of treatment differences

Markers with significant treatment-related effects are summarized in Table 1. Granulocyte Macrophage-Colony Stimulating Factor (GM-CSF), Interleukin-1 beta (IL-1β), Interleukin-4 (IL-4), Interleukin-5 (IL-5), Interleukin-17 (IL-17), and Tumor necrosis factor alpha (TNF-*α*) demonstrated a baseline-by-treatment interaction, in which each 1 MFI unit increase at time-of-treatment, was strongly and positively associated with post-treatment levels in the saline group, a relationship absent or markedly attenuated in the eCB-MSC + HA group. Fractalkine, IL-1β, IL-4, IL-5, Interleukin-18 (IL-18), and TNF-α showed a fragment size-by-treatment interaction, where each 1 mm^2^ increase in fragment size at time-of-treatment was negatively associated with post-treatment levels in the saline group, an association largely abolished in the eCB-MSC + HA treated group. Finally, Interleukin-2 (IL-2) and Interleukin-8 (IL-8) showed significantly lower adjusted post-treatment levels in the eCB-MSC + HA group, independent of baseline levels or fragment size, while Interleukin-13 (IL-13) exhibited a significant baseline effect at the time-of-treatment but no between-group difference in adjusted post-treatment values.

**Table 1:** Synovial fluid cytokine markers with significant treatment-related effects in horses treated with eCB-MSC + HA or saline.

Marker	Interaction p	Saline slope (95% CI)	Saline p	eCB-MSC+HA slope (95% CI)	eCB-MSC+HA p
Baseline-by-treatment interaction					
GM-CSF	<0.001	1.04 (0.79 to 1.28)	<0.001	−0.14 (−0.58 to 0.28)	0.479
IL-1β	<0.001	1.03 (0.86 to 1.18)	<0.001	0.05 (−0.11 to 0.20)	0.544
IL-4	0.006	1.09 (0.97 to 1.21)	<0.001	0.57 (0.24 to 0.89)	0.002
IL-5	0.007	1.20 (1.03 to 1.37)	<0.001	0.63 (0.32 to 0.93)	0.001
IL-17α	0.007	0.50 (0.29 to 0.70)	<0.001	0.05 (−0.15 to 0.25)	0.591
TNF-α	0.045	0.86 (0.59 to 1.13)	<0.001	−0.16 (−1.13 to 0.80)	0.715
Fragment-by-treatment interaction					
Fractalkine	0.026	−0.08 (−0.14 to −0.02)	0.015	0.008 (−0.04 to 0.05)	0.720
IL-1β	0.031	−0.81 (−1.14 to −0.46)	<0.001	−0.35 (−0.67 to −0.02)	0.038
IL-4	<0.001	−1.02 (−1.35 to −0.69)	<0.001	−0.02 (−0.37 to 0.32)	0.878
IL-5	<0.001	−0.60 (−0.83 to −0.38)	<0.001	0.19 (−0.03 to 0.40)	0.113
IL-18	<0.001	−0.86 (−1.26 to −0.46)	<0.001	0.55 (0.23 to 0.87)	0.003
TNF-α	0.040	−0.32 (−0.53 to −0.10)	0.007	−0.02 (−0.26 to 0.21)	0.841
Overall treatment effect (adjusted post-treatment means ± SE)					
IL-2	0.011	17.82 ± 5.47 MFI	–	23.79 ± 5.43 MFI	–
IL-8	0.034	17.3 ± 2.4 MFI	–	25.5 ± 2.2 MFI	–

Each slope shows how strongly one factor predicted a marker’s level, and each interaction tests whether that relationship differed between the two treatment groups. For the *baseline-by-treatment interaction*, the slope reflects how much a marker’s post-treatment level changed for each MFI unit increase in its starting (time-of-treatment) level. For the *osteochondral fragment-by-treatment interaction*, the slope is the expected change in marker level per 1mm^2^ increase in the osteochondral fragment size. Slopes are shown with 95% confidence intervals; the interaction *p*-value tests whether the slopes differed between groups, and each group’s *p*-value tests whether its own slope differed from zero. The final rows (IL-2, IL-8) instead show the overall treatment effect as adjusted post-treatment means ± SE.

**Figure 5:**
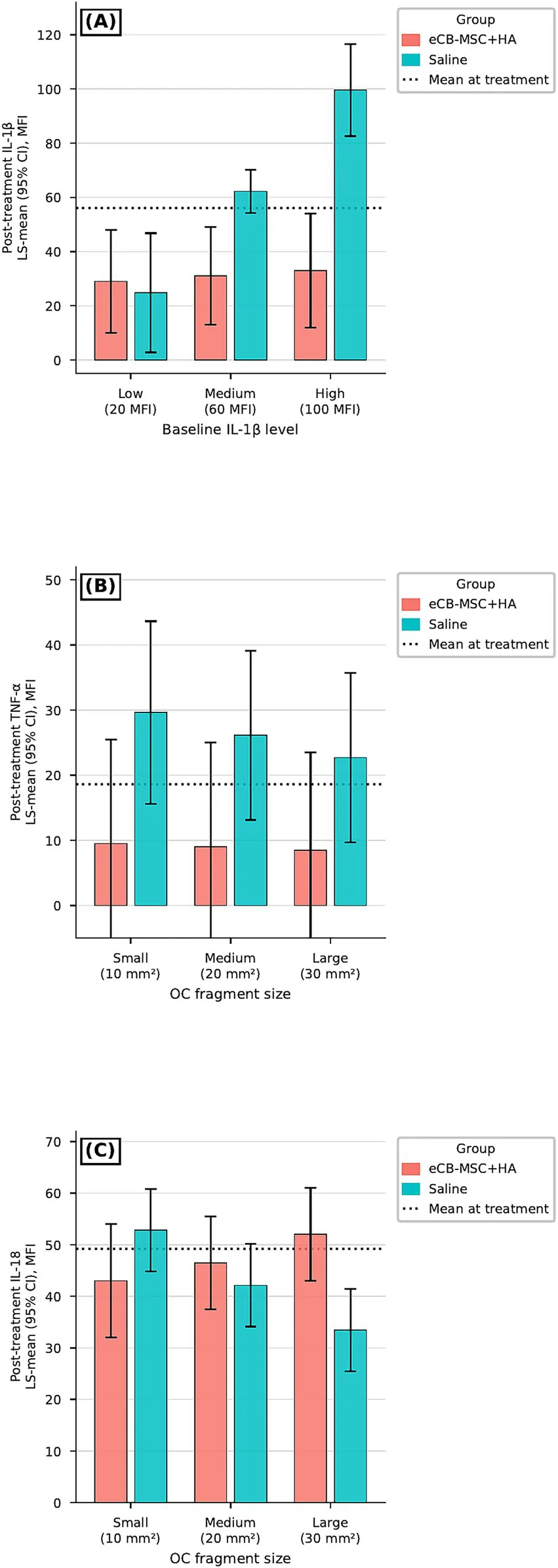
Cytokines with treatment-dependent patterns at post-treatment, shown as LS-means (95% CI). Baseline values are at time-of-treatment. **(A)** IL-1β MFI predicted at low (20 MFI), medium (60 MFI), and high (100 MFI) representative baseline levels, adjusted for mean OC fragment size (27.86 mm^**2**^). The dotted line indicates the mean IL-1β at time-of-treatment (56.1 MFI). Adjusted post-treatment differences (eCB-MSC + HA–Saline): 4.2 MFI at low baseline (*p* = 0.673), −31.19 MFI at medium (*p* = 0.004), and −66.57 MFI at high (*p* = 0.002). **(B)** TNF-*α* MFI predicted at small (10 mm^**2**^), medium (20 mm^**2**^), and large (30 mm^**2**^) OC fragment sizes adjusted for mean baseline TNF-α (18.57 MFI). Adjusted post-treatment differences: −20.14 at small (*p* < 0.001), −17.14 MFI at medium (*p* < 0.001), and −14.19 MFI at large (*p* = 0.002). **(C)** IL-18 MFI predicted at small (10 mm^**2**^), medium (20 mm^**2**^), and large (30 mm^**2**^) OC fragment sizes adjusted for mean baseline IL-18 (49.2 MFI). Adjusted post-treatment differences: −9.82 MFI at small (*p* = 0.085), 4.37 MFI at medium (*p* = 0.406), and 18.56 MFI at large (*p* = 0.006).

Figure 5 illustrates Interleukin 18, Interleukin 1 beta, and tumor necrosis factor alpha, which showed distinct evidence of treatment-related effects. Additional cytokine figures are provided in the Supplementary Figures S4–S9.

### 3.7 Correlation between WORMS scoring and cytokines at time-of-treatment

At time-of-treatment, synovial fluid MFI levels of 11 cytokines showed statistically significant positive correlations with WORMS scoring (R = 0.67–0.91, *p* < 0.05): MCP-1, IFNγ, IL-4, IL-5, IL-2, IL-18, fractalkine, IL-12, FGF-2, IL-8, and GM-CSF; Supplementary Table S4 and Figure S9.

## 4 Discussion

The results of this randomized study support the hypothesis that intra-articular administration of pooled cryopreserved non-activated eCB-MSCs combined with HA is safe and biologically active in a surgically induced equine fetlock OA model. Horses treated with eCB-MSCs+HA showed greater clinical improvement, differences in MRI-detected joint structure, and modulation of key inflammatory and anti-inflammatory mediators compared with saline-treated controls, providing preliminary converging evidence for both symptomatic and potential disease-modifying effects.

Importantly, the therapeutic response was not uniform and depended on time-of-treatment disease activity and fragment size, highlighting the relevance of phenotype-specific OA biology when interpreting MSC efficacy.

No clinically relevant adverse joint reactions were observed following intra-articular eCB-MSC + HA administration. Joint effusion scores, total nucleated cell counts, and synovial protein concentrations remained comparable between groups, supporting the local safety of pooled, cryopreserved allogeneic eCB-MSCs combined with HA. These findings align with prior equine studies demonstrating that while MSC administration may induce transient inflammatory changes, these responses are typically mild, self-limiting, and not associated with sustained clinical deterioration (40, 42, 51).

### 4.1 Clinical outcomes

Both objective and subjective lameness assessments showed significantly greater improvement after 6 weeks of treatment in horses treated with eCB-MSC + HA than in those receiving saline. Although the inertial-sensor measure is continuous and carries greater statistical power than an ordinal scale, the two modalities were concordant, supporting a genuine treatment-related reduction in lameness.

These findings are consistent with previous studies in which intra-articular MSC therapy, across multiple cell sources, improved clinical outcomes in horses with OC fragment models or naturally occurring fetlock osteoarthritis (22, 23, 40, 52).

A strong correlation between subjective and objective lameness measures was observed at both time-of-treatment and post-treatment time points. This contrasts with earlier reports describing weaker agreement between assessment modalities (53, 54). The use of a modified AAEP grading scale with 0.5 increments likely contributed to improved sensitivity, enabling detection of subtle but clinically relevant changes that may not be captured by whole-grade scoring systems (40). When combined with objective gait analysis, this semi-quantitative approach improves resolution and may better reflect clinical improvement in experimental OA studies.

Despite these findings, lameness evaluation should not rely solely on limb-specific or straight-line assessments. Subtle alterations in behavior (55) or axial skeletal symmetry (56) may precede detectable asymmetries in head or pelvic movement, and examiner subjectivity remains an inherent limitation of clinical scoring systems. For future studies, incorporation of a more comprehensive lameness examination protocol—including lunging in both directions and assessment on multiple surfaces—is recommended, as straight-line evaluation alone may fail to reliably distinguish lame from sound horses (57).

Flexion testing has previously been shown to enhance differentiation between treatment groups in MSC studies (58). In the present study, flexion tests were not consistently performed at all time points and were therefore excluded from formal analysis. However, emerging data from this group suggest that grading lameness after flexion using a 0–5 scale with 0.5 increments may improve standardization and can facilitate direct comparison with objective gait analysis outputs following flexion, potentially strengthening outcome assessment in future OA trials (40).

### 4.2 MRI-detected structural changes

In parallel with efforts to standardize clinical outcome measures, the inclusion of high-field MRI with the WORMS system provided complementary structural information in the equine fetlock OC fragment model when compared with conventional radiography. In human OA research, MRI is widely regarded as the gold-standard imaging modality for detecting both acute and chronic osteoarthritic changes (59–61), and semiquantitative scoring systems such as WORMS have been instrumental in improving interpretability and reproducibility of imaging outcomes under OARSI recommendations (62). Consistent with these principles, and although MRI is not routinely available for clinical diagnosis of OA in horses, several investigators have emphasized the importance of standardized MRI-based scoring systems for equine OA research (13, 37, 38). In the present study, a WORMS-based scoring system, modified from a previous investigation (38) and adapted specifically for the metacarpophalangeal joint, was applied to support ongoing standardization efforts and to enhance structural outcome assessment; importantly, MRI revealed differences in structural outcomes between the eCB-MSC + HA and saline groups that were not apparent on radiography or arthroscopy. Given the absence of corroborating findings on these modalities, the limited sample size, and variability in lesion characteristics, these MRI-based differences should be interpreted with caution.

Reliable detection of early osteophytosis, particularly grade 1 changes, remains challenging using conventional imaging modalities such as radiography in both humans and horses. In this context, MRI has demonstrated superior sensitivity for identifying early osteoarthritic structural changes (29, 63). Consistent with previous reports (11), the present study demonstrated development of a “kissing lesion” on the opposing metacarpal condyle in addition to the surgically induced subchondral defect on the proximal phalanx. This opposing lesion resulted in focal subchondral bone injury and likely contributed to the bone marrow–like lesions observed adjacent to this region.

**Figure 6:**
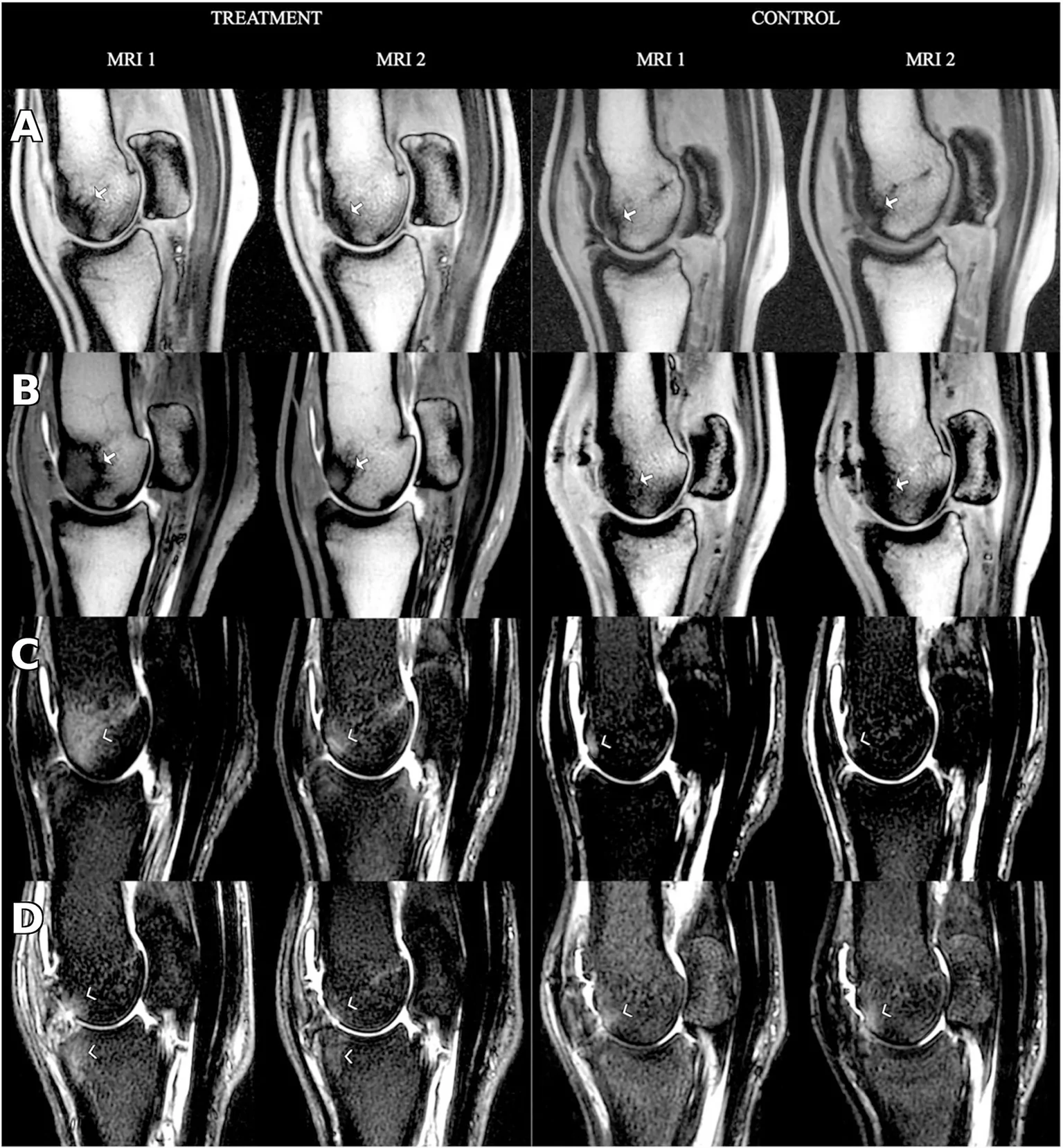
Treatment vs. control horses MRI comparisons. Upper 2 rows show combined images from sagittal T1 FSPGR **(A)** and T2* FGRE **(B)** sequences from horses in the eCB-MSC + HA vs. saline groups, focusing on the most significant low signal changes (white arrows). Lower 2 rows **(C, D)** show sagittal STIR sequences from horses in the eCB-MSC + HA vs. saline groups, focusing on the most significant high signal changes (white arrow heads).

Bone marrow lesions identified on T1 fat-suppressed fast spoiled gradient-recalled (FSPGR), T2* fast gradient-recalled echo (FGRE), and Short Tau Inversion Recovery (STIR) sequences (Figure 6) were consistent with findings reported in prior equine OC fragment OA models (13, 37). Although such lesions have been described as transient during the early stages of OA in both humans and horses and may partially resolve over time (64), their presence in combination with early osteophytosis and subchondral bone abnormalities supports the diagnosis of an early osteoarthritic process. This constellation of MRI findings may represent a clinically relevant window for intervention, during which disease-modifying strategies could alter subsequent joint degeneration.

Another articular structure with significant impact in OA pathogenesis is the synovium. Synovitis has been recognized as an early pathological change and may also contribute to OA progression, depending on the structural and metabolic severity as well as disease duration (65, 66). Our findings show that horses treated with eCB-MSC + HA exhibited a 0.6 mm reduction in synovial thickness after treatment compared with saline controls. Although this effect may be partly attributable to HA, a recent human randomized controlled trial reported significant improvement in synovitis at 6 months following UC-MSC treatment compared with HA and placebo controls (67).

Taken together, these MRI-based findings suggest that eCB-MSC + HA may attenuate early structural progression of OA in this model in addition to improving clinical signs of disease.

### 4.3 Synovial fluid cytokines and immunomodulatory effects

Beyond clinical and structural outcomes, this study evaluated synovial fluid cytokines to test the hypothesis that eCB-MSC + HA treatment modulates the inflammatory and immunologic environment of the osteoarthritic joint. Among the classical inflammatory mediators, IL-1β and TNF-*α* are well-established drivers of OA progression through their roles in synovial inflammation and cartilage catabolism (68). Although arthropathies differ in etiology, the OC fragment model used here represents a controlled model of focal joint injury rather than the full spectrum of naturally occurring PTOA. Nevertheless, it captures selected inflammatory features of injury-associated joint disease. Evidence for this pattern in naturally occurring disease comes from a 4-year longitudinal study of Standardbred racehorses examining spontaneous fetlock PTOA, in which IL-1β, TNF-α, and IL-6 increased progressively over time (69).

Consistent with these findings, saline-treated joints in the present study showed post-treatment increases in IL-1β and TNF-α that were positively associated with time-of-treatment values, suggesting persistence or amplification of inflammation. In contrast, eCB-MSC + HA-treated joints maintained low values of both cytokines across a wide range of time-of-treatment values, consistent with a targeted immunomodulatory effect rather than uniform cytokine suppression. Although IL-6 did not reach statistical significance, post-treatment values in eCB-MSC + HA-treated horses were approximately half those of saline group, suggesting a similar directional effect that may have been underpowered in this cohort.

IL-8, a chemokine linked to neutrophil recruitment and inflammatory amplification (70, 71), has been shown to behave variably across MSC sources and experimental systems (72, 73). In naturally occurring OA, IL-8 may remain persistently elevated, contributing to sustained inflammation (74). In the present study, IL-8 showed an overall treatment effect, with lower adjusted post-treatment values in eCB-MSC + HA-treated joints than in the saline group, consistent with attenuation of chemokine-associated inflammatory signaling.

In addition to these classical mediators, MSCs appear to influence macrophage-associated cytokines such as GM-CSF, which promotes M1 macrophage differentiation and inflammatory signaling in OA (75). In saline-treated joints, higher time-of-treatment GM-CSF values were associated with further post-treatment increases, consistent with persistent inflammation. In contrast, GM-CSF values in eCB-MSC + HA-treated joints decreased or remained stable regardless of time-of-treatment values, possibly reflecting a shift toward a less pro-inflammatory macrophage environment, although macrophage phenotype was not directly assessed.

At the level of adaptive immunity, MSCs influence T helper cell polarization, including Th1, Th2, and Th17 pathways, in response to the local cytokine milieu (76, 77). Th2-associated cytokines such as IL-4 and IL-5 are of particular interest due to their anti-inflammatory properties and potential protective roles in OA (78, 79). In this study, increasing fragment size in saline treated joints was associated with declining IL-4 and IL-5 values, whereas eCB-MSC + HA-treated joints maintained relatively stable values across fragment severities. Although eCB-MSC + HA treatment did not induce overt increases in these cytokines, their preservation with increasing joint injury could suggest that Th2-associated signaling was maintained as OA severity progressed (80, 81).

IL-2, primarily secreted by Th1 cells, is another cytokine traditionally viewed as pro-inflammatory; however, its role in OA is increasingly recognized as context-dependent, particularly given its importance in regulatory T-cell homeostasis and Th17/Treg balance (82–86). In the present study, IL-2 showed an overall treatment effect, with lower adjusted post-treatment values in eCB-MSC + HA-treated joints than in saline treated ones. Time-of-treatment IL-2 values remained positively associated with post-treatment concentrations, and an overall fragment effect was also observed, suggesting that both pre-existing immune activity and lesion severity contributed to IL-2 expression after treatment.

Finally, IL-18, a member of the IL-1 cytokine family, plays a dual role in inflammation and immune regulation, influencing both Th1 and Th2 responses depending on the cytokine context (76, 77, 87, 88). A significant fragment–treatment interaction was observed, whereby saline-treated joints with large fragments exhibited a marked reduction in IL-18 values, while eCB-MSC + HA-treated joints maintained significantly higher values. When considered alongside the Th2-associated cytokine patterns, these findings raise the hypothesis that MSCs may modulate the adaptive immune environment rather than uniformly inhibiting inflammation.

These pathway-level interpretations, including effects on macrophage polarization and T-helper-cell balance, are inferred from synovial fluid cytokine patterns rather than from direct cellular assessment, and should be regarded as hypothesis-generating, thus requiring confirmation in dedicated mechanistic equine studies.

The strong correlations between time-of-treatment WORMS and multiple synovial fluid cytokines (Supplementary Table S4 and Supplimentary Figure S9) are consistent with a link between structural disease burden and joint inflammation in this model. These associations provide a biologically relevant framework for interpreting the immunomodulatory effects observed following eCB-MSC + HA treatment.

### 4.4 Study limitations

Although the primary aim of this study was not to revalidate the metacarpophalangeal joint P1 OC fragment model, variability in OC fragment size created using the customized 55° wedge represents a potential limitation. Fragment size varied (range, 6.9–65 mm^2^; mean ± SD, 26.1 ± 20.0 mm^2^; median, 18.4 mm^2^) widely, and one horse developed a surgically induced short parasagittal P1 fracture (Supplementary Figure S3). As the clinical presentation of this horse did not differ substantially from that of the remaining cohort, and the fracture was only recognized later when scoring the first MRI images, the horse was not excluded; however, the variability in fragment size highlights practical challenges inherent to the model, including differences in P1 contour between horses, external limb conformation influencing wedge positioning, subtle millimetric adjustments required to engage the articular surface, and unavoidable variation in applied force during fragment creation. Although earlier carpal OC fragment models described more consistent 8-mm-wide fragments (89), subsequent studies have reported difficulties standardizing fragment size and have raised concerns about its influence on OA severity and progression (37). Similar limitations have not been previously emphasized in fetlock P1 OC fragment models (11–13). In the present study, fragment size showed a significant baseline (time-of-treatment) effect on several cytokines. Although this association was statistically clear, the small sample size precludes defining its biological significance or a link to OA severity. This variability nonetheless likely reflects the clinical reality of naturally occurring OA, where lesion size and morphology are inherently diverse. Importantly, treatment-related differences persisted across a wide range of fragment sizes, consistent with eCB-MSC + HA exerting immunomodulatory and potential disease-modifying effects.

Broader limitations include the small sample size, the short follow-up, and the fact that between-group structural differences were detected on MRI but not on radiography or arthroscopy. These factors constrain conclusions regarding disease modification, which require confirmation in larger studies with longer-term follow-up.

### 4.5 Conclusion

In conclusion, this randomized study demonstrated that intra-articular administration of pooled cryopreserved eCB-MSCs combined with HA is safe and associated with improved clinical outcomes, attenuation of MRI-detected structural changes, and modulation of synovial pro- and anti-inflammatory mediators in a surgically induced equine fetlock osteoarthritis model. Treatment responses varied according to baseline (time-of-treatment) inflammatory activity and osteochondral fragment size, suggesting that eCB-MSCs combined with HA exert context-dependent immunomodulatory effects rather than uniform suppression of inflammation. The observed relationships among lameness, MRI findings, and synovial cytokine profiles further support the integration of clinical, imaging, and molecular biomarkers when evaluating potential disease-modifying osteoarthritis therapies. Collectively, these findings support the potential of eCB-MSC-based therapies as symptom- and possibly disease- modifying treatments for early post-traumatic osteoarthritis. Because the structural evidence rested on MRI findings alone, with a small sample size and short follow-up time, the disease-modifying effects should be considered preliminary and confirmed in larger studies with longer-term follow-up.

## Data Availability

The raw data supporting the conclusions of this article will be made available by the authors, without undue reservation.
