# Supplementary material for: Cord blood mesenchymal stromal cells combined with hyaluronic acid show symptom-modifying and potential disease-modifying effects in an equine osteoarthritis fetlock chip model
Source: Front Vet Sci. 2026 Aug 6;13:1896354. doi: 10.3389/fvets.2026.1896354 (PMC13491432; doi:10.3389/fvets.2026.1896354)
Supplement: Supplementary file 1 [file Supplementary_file_1.docx]

Supplementary Material

# 1 Supplementary Tables

**Table S1. Adapted lameness scoring system for the distal limb including flexion test response.1 PFT (Passive Flexion Test).**

|  | **Grade** | **Interpretation** |
| --- | --- | --- |
| **Lameness Grading** | 0 | Lameness not perceptible under any circumstances. |
|  | 0.5 | Lameness may only be barely noticeable after PFT but not without it. |
|  | 1 | Slight lameness, difficult to observe at a walk or when trotting in a straight line but consistently apparent under certain circumstances (e.g. circling, inclines, hard surface, etc.). Lameness will barely be obvious few steps after PFT. |
|  | 1.5 | Mild intermittent lameness at trot, lameness is mildly increased after PFT but soon will return to baseline. |
|  | 2 | Mild to moderate lameness difficult to observe at a walk or when trotting in a straight line but consistently apparent under certain circumstances (e.g. circling, inclines, hard surface, etc.). Lameness may be mildly increased after PFT only while going but not coming back. |
|  | 2.5 | Moderate lameness at trot, lameness may be moderately increased after PFT while going and some steps coming back. |
|  | 3 | Moderate to marked lameness consistently observable at a trot under all circumstances, with moderate increase lameness after PFT and mild reluctance to it, horse may go and come back lamer. |
|  | 3.5 | Marked lameness at trot and noticeable at walk, the horse will allow PFT but may only resist 20–30 seconds and lameness will be markedly increased going and coming back. |
|  | 4 | Severe lameness seen at walk, may be reluctant to trot, horse may allow PFT but soon will avoid it and lameness will severely be increased afterwards. |
|  | 4.5 | Severe lameness seen at walk, horse allows light palpation but no passive flexion. |
|  | 5 | Lameness produces minimal weight bearing in motion and/or at rest or a complete inability to move, with the horse completely reluctant to any manipulation of the affected area. |

Table S2. Magnetic Resonance Imaging parameters

|  | **FOV  mm** | **Slice / Gap  mm** | **TE  ms** | **TR  ms** | **TI  ms** | **FA** | **NEX** | **Matrix** | **Pixel size  mm** | **Imaging  Option** |
| --- | --- | --- | --- | --- | --- | --- | --- | --- | --- | --- |
| **Sag T2-STIR** | 190 | 3/1 | 41.52 | 3173 to 5775 | 150 | 180 | 3 | 256×256 | 0.7×0.7 | ZIP 512 |
| **Sag T2* FGRE** | 190 | 3/−1.5ov | 2.4 | 6.7 | — | 30 | 2 | 256×256 | 0.7×0.7 | ZIP2 & 512 |
| **Isotropic 3D Dor T1 FSPGR**  **Fat Sat (SPECIAL)** | 190 | 1.4/−0.7 | 4.2 | 8.6 | 25 | 20 | 3 | 256×256 | 0.7×0.7 | ZIP 2 |
| **Dor T2* FGRE** | 190 | 3/−1.5ov | 2.3 | 6.5 | — | 30 | 2 | 256×256 | 0.7×0.7 | ZIP2 & 512 |
| **Tran T2* FGRE** | 170 | 3/−1.5ov | 2.8 | 8.2 | — | 30 | 2 | 224×192 | 0.8×0.9 | ZIP2 & 512 |
| **Tran STIR** | 170 | 3/0 | 40 | 3899 to 5206 | — | 150 | 3 | 256×256 | 0.7×0.7 | ZIP 512 |

**Table S3. Adapted metacarpophalangeal joint modified whole organ magnetic resonance score system (WORMS).^2^**

| **Parameter** | **0** | **1** | **2** | **3** | **4** |
| --- | --- | --- | --- | --- | --- |
| **High signal bone lesions (size)** | None | < 25% of the region | 25–50% of the region | > 50% of the region | — |
| **High signal bone lesions (intensity)** | None | Mild | Moderate | Marked | — |
| **Low signal bone lesions** | < 10% of the region | 10–25% of the region | 26–50% of region | > 50% of region | — |
| **Osteophyte formation, size of the largest in the region** | None | < 1 mm | 1–2 mm | > 2 mm | — |
| **Cartilage signal abnormality** | None | Single lesion < 5 mm | Single lesion 5–10 mm or multiple lesions < 5 mm | Single lesion > 10 mm or multiple lesions 5–10 mm | — |
| **Subchondral bone irregularity** | Smooth and regular chondro-osseous junction | Mild subchondral plate irregularity | Marked subchondral plate irregularity with intact trabecular bone | Bone irregularity extending to the trabecular bone with preservation of some trabecular pattern | Cyst-like formation |
| **Joint effusion** | Small expected amount of fluid | Mild increase | Moderate increase | Severe increase | — |
| **Capsule/synovium thickness (tissue of moderate signal intensity protruding into the hyperintense synovial fluid)** | Normal | Mild | Moderate | Severe | — |

Table S4. Pearson correlation coefficients between synovial fluid cytokine/chemokine mean fluorescence intensities and Whole-Organ Magnetic Resonance Imaging Score (WORMS) at time-of-treatment, ranked by R value. Bold entries indicate statistical significance (p < 0.05)

| **Rank** | **Marker** | **R** | **p-value** |
| --- | --- | --- | --- |
| **1** | **MCP-1** | **0.906** | **0.0001** |
| **2** | **IFNγ** | **0.798** | **0.0032** |
| **3** | **IL-5** | **0.749** | **0.008** |
| **4** | **IL-4** | **0.755** | **0.0073** |
| **5** | **IL-2** | **0.729** | **0.0108** |
| **6** | **IL-18** | **0.719** | **0.0126** |
| **7** | **Fractalkine** | **0.710** | **0.0145** |
| **8** | **IL-12** | **0.692** | **0.0183** |
| **9** | **FGF-2** | **0.677** | **0.022** |
| **10** | **IL-8** | **0.676** | **0.0223** |
| **11** | **GM-CSF** | **0.668** | **0.0248** |
| 12 | IP-10 | 0.593 | 0.0925 |
| 13 | IL-1α | 0.570 | 0.0671 |
| 14 | IL-1β | 0.542 | 0.0848 |
| 15 | TNF-α | 0.484 | 0.1314 |
| 16 | IL-17A | 0.442 | 0.1738 |
| 17 | Eotaxin | 0.409 | 0.2111 |
| 18 | PGE2 | 0.347 | 0.296 |
| 19 | G-CSF | 0.331 | 0.3496 |
| 20 | IL-13 | 0.324 | 0.3307 |
| 21 | IL-10 | 0.163 | 0.6526 |
| 22 | GRO | 0.107 | 0.7531 |
| 23 | RANTES | −0.123 | 0.7534 |
| 24 | IL-6 | −0.053 | 0.8781 |

## 2. Supplementary Figures

**I**

**II**

**III**

**IV**

**V**

**VI**

**VII**

**VIII**

**Lateral**

**Medial**

**1**

**2**

**3**

**4**

**5**

**6**

**7**

**8**

**9**

**10**

**11**

**13**

**12**

Figure S1. Adapted metacarpophalangeal joint regions for imaging scoring.^2^

WORMS scoring regions are displayed in Roman numerals, and radiography scoring regions in Arabic numerals. (Image courtesy of A. Merchan)

Figure S2. Comparative dorsolateral-palmaromedial oblique radiographic views from 2 horses from each group at baseline and discharge after 4 months.

The most representative dorsomedial osteophyte formation samples (red circles) were selected within each group.

Figure S3. MRI images from horse with iatrogenic fracture.

Right front fetlock transverse T1 FSPGR (left) and a 3D dorsal T1 FSPGR FS (right) sequences from the horse who sustained a unicortical parasagittal P1 fracture (red circles) following the osteochondral fragment creation.

Figure S4. Adjusted GM-CSF mean fluorescence intensity (MFI) least squares mean (95% CI) post-treatment between groups, stratified by baseline GM-CSF MFI (10, 15, 20 units).

The horizontal dotted line indicates the mean baseline GM-CSF MFI (14.8 MFI). The adjusted Treatment–Control difference was 1.57 at baseline 10 MFI (p = 0.529), –4.54 at baseline 15 MFI (p = 0.055), and –10.66 at baseline 20 MFI (p = 0.015).

Figure S5. IL-2 mean fluorescence intensity (MFI) least-squares means (95% CI) post-treatment between groups by osteochondral fragment size (10, 20, 30 mm²).

The horizontal dotted line represents the mean IL-2 baseline of 19.5 MFI units. At 10 mm², the estimated between-group difference was –12.6 MFI units (p = 0.016); at 20 mm², –6.7 MFI units (p = 0.054); and at 30 mm², –0.9 MFI units (p = 0.778).

**Figure S6. IL-4 mean fluorescence intensity (MFI) least-squares means (95% CI) post-treatment between groups by osteochondral fragment size (10, 20, 30 mm²).**

The horizontal dotted line represents the mean IL-4 baseline of 56.6 MFI units. At 10 mm², the estimated between-group difference was –27 MFI units (p = 0.001); at 20 mm², –17 MFI units (p = 0.011); and at 30 mm², –7 MFI units (p = 0.216).

Figure S7. IL-5 mean fluorescence intensity (MFI) least-squares means (95% CI) post-treatment between groups by osteochondral fragment size (10, 20, 30 mm²).

The horizontal dotted line represents the mean IL-5 baseline of 38.6 MFI units. At 10 mm², the estimated between-group difference was –15.2 MFI units (p = 0.002); at 20 mm², –7.3 MFI units (p = 0.006); and at 30 mm², 0.6 MFI units (p = 0.86).

Figure S8. Adjusted IL-17a mean fluorescence intensity (MFI) least squares means (95% CI) post-treatment between groups, stratified by baseline IL-17a MFI (9, 10.5, 12.5 units).

The horizontal dotted line indicates the mean baseline IL-17a MFI (12.14 MFI). The adjusted Treatment–Control difference was 0.52 at baseline 9 MFI (p = 0.384), –0.14 at baseline 10.5 MFI (p = 0.745), and –1.05 at baseline 12.5 MFI (p = 0.020).

Figure S9. Pearson correlation coefficients between baseline (before treatment) synovial fluid cytokine/chemokine mean fluorescence intensities and Whole-Organ Magnetic Resonance Imaging Score (WORMS).

Several analytes showed strong positive correlations with WORMS, including MCP-1, IFN-γ, IL-5, IL-4, IL-2, IL-18, and fractalkine (P < 0.05). Additional moderate positive correlations were observed for IL-12, FGF-2, IL-8, and GM-CSF (P < 0.05). Several cytokines showed moderate correlation coefficients that did not reach statistical significance, likely reflecting the limited sample size, whereas GRO, RANTES, and IL-6 showed negligible associations with WORMS. Positive correlation coefficients indicate that higher cytokine values are associated with increasing structural disease burden.
